# Cell wall biosynthesis impairment affects the budding lifespan of the *Saccharomyces cerevisiae* yeast

**DOI:** 10.1007/s10522-017-9740-6

**Published:** 2017-11-30

**Authors:** Mateusz Molon, Olga Woznicka, Jacek Zebrowski

**Affiliations:** 1 Department of Biochemistry and Cell Biology, University of Rzeszow, Zelwerowicza 4, 35-601 Rzeszow, Poland; 2 Department of Cell Biology and Imaging, Institute of Zoology, Jagiellonian University, Krakow, Poland; 3 Department of Plant Physiology, Institute of Biotechnology, University of Rzeszow, Rzeszow, Poland

**Keywords:** Yeast, Budding lifespan, Aging, Cell wall, AFM, SEM, TEM

## Abstract

The *Saccharomyces cerevisiae* yeast is one of the most widely used model in studies of cellular and organismal biology, including as aging and proliferation. Although several constraints of aging and budding lifespan have been identified, these processes have not yet been fully understood. Previous studies of aging in yeast have focused mostly on the molecular basics of the underlying mechanisms, while physical aspects, particularly those related to the cell wall, were rather neglected. In this paper, we examine for the first time, to our knowledge, the impact of cell wall biosynthesis disturbances on the lifespan in the budding yeast. We have used a set of cell wall mutants, including *knr4Δ*, *cts1Δ*, *chs3Δ*, *fks1Δ* and *mnn9Δ*, which affect biosynthesis of all major cell wall compounds. Our results indicated that impairment of chitin biosynthesis and cell wall protein mannosylation reduced the budding lifespan, while disruption in the 1,3-β-glucan synthase activity had no adverse effect on that parameter. The impact varied in the severity and the most notable effect was observed for the *mnn9Δ* mutant. What was interesting, in the case of the dysfunction of the Knr4 protein playing the role of the transcriptional regulator of cell wall chitin and glucan synthesis, the lifespan increased significantly. We also report the phenotypic characteristics of cell wall-associated mutants as revealed by imaging of the cell wall using transmission electron microscopy, scanning electron microscopy and atomic force microscopy. In addition, our findings support the conviction that achievement of the state of hypertrophy may not be the only factor that determines the budding lifespan.

## Introduction

*Saccharomyces cerevisiae* is one of the most widely used model organism in the research of cellular processes, including aging and proliferation. Compared to mammalian cells, the budding fungi show specifically asymmetric cytokinesis, close mitosis and the presence of the cell wall, among which the latter may have considerable effect on aging (Lippuner et al. 2014; Steinkraus et al. 2008). It has been reported that some cell wall properties, including composition, size and surface wrinkling, may be age-associated (Cabib et al. 1997; Egilmez et al. 1990; Powell et al. 2000). Cell wall plays a multifunctional role in yeast’s living processes (Gow et al. 2017; Lesage and Bussey 2006) and its synthesis, maintenance and remodelling is controlled by a large (above 1200) amount of genes (de Groot et al. 2001). The wall providing a relatively rigid envelope to the cell within the plasmalemma is essential for fungal cell growth, reproduction and interaction with environment. Particularly, it controls the cell’s shape and growth rate, ensuring protection against external mechanical factors and internal osmotic pressure (Gow et al. 2017; Lesage and Bussey 2006). The wall polysaccharides provide the scaffold for surface glycoproteins which contribute to the adhesive wall properties and reduce wall permeability to large molecules, particularly wall digestive enzymes. Cell wall is a highly dynamic structure whose chemical composition and polymer interlinkage pattern respond to developmental changes and environmental cues. The *Saccharomyces cerevisiae* yeast cell wall is composed of external layer rich in *O*- and *N*-mannosylated proteins, imaged in the electron microscope as an electron dense part, and the layer adjacent to plasma membrane composed mostly of β-1,3 glucans branched to some extent by β-1,6 bonds and a much smaller amount of chitin which interlinks the glucan polymers and other wall compounds into a load bearing-matrix (Klis et al. 2006; Smith et al. 2000).

A number of data indicate direct involvement of the cell wall in the reproductive processes, including budding (Cabib et al. 2001; Roncero and Sanchez 2010). Separation of mother and daughter cells in the budding yeast is essential for the organism’s proliferation. This is preceded by formation of septa, a process closely linked to cytokinesis and division of nucleus. Following actomyosin ring contraction, a primary septum is formed which is built mainly of chitin fibres synthesised by chitin synthase II with Chs2p as a catalytic unit. Upon completion of this process, the secondary septum is laid down from both mother and daughter sides through the deposition of glucans and chitin as the major compounds. Synthesis of β-1,3 glucans is driven by glucan synthase Fks1 (Cabib et al. 2001; Lesage and Bussey 2006; Lesage et al. 2005) while the deposition of chitin requires chitin synthase Chs3 (Cabib et al. 2001; Cabib and Schmidt 2003; Ortiz and Novick 2006; Schmidt et al. 2002; Ziman et al. 1998). To enable physical separation of mother and daughter cells, both primary and secondary septa layers must undergo destruction and remodelling that is performed by digestive enzymes, including chitinase (Cts1) (Kuranda and Robbins 1991) and glucanases/glucosyltransferases. Formation and disassembly of septa employs, apart from some specific enzymatic complexes, a generally similar machinery that is used for synthesis and remodelling of the lateral cell wall. Thus, defects in the biosynthesis of particular cell wall compounds may affect septation and therefore the budding process in yeast cells. However, septation may take place even when either the primary septum is not formed or actomyosin ring contraction does not occur, and an alternative remedial septum, which is a form of the secondary septum, allows for performing separation of the mother and daughter cells (Cabib and Schmidt 2003; Tolliday et al. 2003). The budding process must undergo a coordinated control in respect of maintaining cell wall integrity, particularly in the region of the mother–bud neck. To meet this requirement and to prevent local cell surface extension, the chitin ring formed at the neck is bound to β-1,3-glucan in the cell wall (Arroyo and Arroyo 2013).

In 1959, Mortimer and Johnston were the first to discover that a single yeast cell (“mother”) has limited reproductive potential (budding lifespan) (Mortimer and Johnston 1959). Earlier observation suggested that the new bud is never formed at the site of the older bud (Barton 1950). Therefore, the number of buds (“daughters”) formed by a single cell defines the reproductive age. The first calculations predicted that yeast cannot produce more than 100 buds (Bartholomew and Mittwer 1953). The first estimations, technically still limited, allowed for observing 23 bud scars at the most (Barton 1950). Recent studies showed that in the case of some mutants, the “mother” cell can produce more than 70 buds (Molon et al. 2016). To this day, several factors have been identified as potential aging constraints (reviewed by Steinkraus et al. 2008), including accumulation of extrachromosomal rDNA circles (ERCs) (Sinclair and Guarente 1997), oxidative damage of protein (Aguilaniu et al. 2003) or thermal aggregates (Erjavec et al. 2007; Molon and Zadrag-Tecza 2016a). They were taken into account in various proposed hypotheses; however, none of them explains definitively the phenomenon of the reproductive potential limit.

Cell wall involvement in longevity of fungi is still little understood. This may be putatively associated with the role of the wall in the control of volumetric growth and morphogenesis. One of the consequences of the choice of budding as the method of asexual reproduction is the continuous increase in cell volume. During subsequent doublings, the cell increases in size and changes its shape (Bartholomew and Mittwer 1953; Molon and Zadrag-Tecza 2016b; Powell et al. 2000; Zadrag-Tecza et al. 2009), which is to a great extent controlled by cell wall properties.

In this paper we present, for the first time to our knowledge, the effect of disturbances in cell wall biosynthesis on the budding lifespan of yeast. The obtained data emphasize the role of cell wall status, including possibly cell wall integrity, on the budding lifespan of yeast. We have explored mutations affecting all main cell wall compounds, including 1,3-β-glucans, chitin and mannoproteins. Additionally, we show morphological changes in the cell wall at the ultrastructural level revealed by scanning electron microscopy (SEM) and transmission electron microscopy (TEM) as well as atomic force microscopy (AFM) imaging. Taking into account the widespread contribution of cell wall characteristics to the growth, reproduction and sensing environment, linking the cell wall features to longevity may help better understand the complexity of the determinants of aging in *S. cerevisiae*.

## Materials and methods

### Yeast strains

The strains used in this study are given in Table 1.

**Table 1:** Strains used in this study

Strain	Genotype	Source
BY4741	*MATa his3 leu2 met15 ura3*	EUROSCARF
*chs3Δ*	*MATa his3 leu2 met15 ura3 YBR023C::kanMX4*	EUROSCARF
*cts1Δ*	*MATa his3 leu2 met15 ura3 YBR023C::kanMX4*	EUROSCARF
*fks1Δ*	*MATa his3 leu2 met15 ura3YLR342* *W::kanMX4*	EUROSCARF
*knr4Δ*	*MATa his3 leu2 met15 ura3 YGR229C::kanMX4*	EUROSCARF
*mnn9Δ*	*MATa his3 leu2 met15 ura3 YPL050C::kanMX4*	EUROSCARF

### Growth conditions

Yeast cells were grown in a standard liquid YPD medium (1% Difco Yeast Extract, 1% Yeast Bacto-Peptone, 2% glucose) on a rotary shaker at 150 rpm, or on a solid YPD medium containing 2% agar. The experiments were carried out at the temperature of 28 °C.

### Determination of budding lifespan

Yeast budding lifespan was determined according to previously described procedure (Wawryn et al. 1999). Yeast cultures were grown in a rich YPD medium (1% bacto-peptone, 1% yeast extract, 2% glucose, 2% agar) to the log phase. Five microliter aliquots of each culture was dropped on separate YPD plates. Forty single cells were micromanipulated for each experiment. Analysis were determined by micromanipulation using an optical microscope Nikon Eclipse E200 with attached micromanipulator. The number of buds formed by each cell was used to determine its reproductive potential. During the manipulation, the plates were kept at 28 °C for 16 h and at 4 °C during the night. The data represent the mean values from three separate experiments.

### Scanning electron microscopy preparation

For scanning electron microscopy (SEM) the samples were fixed in 2.5% (v/v) glutaraldehyde GLU in 0.1 M phosphate buffered saline (PBS) by 2 h, rinsed with PBS 2 × 10 min and dehydrated in graded alcohols. Finally, it was placed in transitional liquid (100% acetone) and transferred to the Critical Point Drier (CPD E3000/E3100 Quorum Technologies). Then it was coated with gold using JFC-1100E Ion sputter (Jeol). For coating, the materials were placed on the holder with conductive carbon adhesive tabs (Electron Microscopy Sciences). Morphological characters were analyzed by means of Scanning Electron Microscope (JSM-5410).

### Transmission electron microscopy preparation

Samples were fixed in a primary fixative 2% (v/v) glutaraldehyde, 2.5% (v/v) paraformaldehyde in a cacodylic buffer and were postfixed for 2 h in 2% OsO_4_. Next, samples were dehydrated in an alcohol series and twice in propylene oxide before being embedded in Poly/bed 812 resin (Polysciences). Embedded samples were sectioned with a Reichert Ultracut and then observed by using the transmission electron microscopy. For determination of yeast cell wall thickness images were captured with the microscope equipped with the TVIPS digital camera. Cell thickness was measured using the EM MENU4 software.

### Atomic force microscopy

*Saccharomyces cerevisiae* cells were collected at the exponential growth stage, three times washed in PBS buffer and deposited on a microscopic cover glass. Subsequently, they were dried under N_2_ atmosphere at ambient temperature. AFM topographical imaging was performed in air in the PeakForce Tapping mode using the BioScope Catalyst II system with the Nanoscope V controller (Veeco Instruments, Santa Barbara, CA, US) and silicon nitride MLCT probes (Bruker, Camarillo, CA). The Height and PeakForce Error images were obtained at the scan rate of 0.33 Hz and with 512 pixels per line using the Nanoscope (1.40 v.5, Bruker) software and the ScanAsyst algorithm for the optimization of the gain and setpoint parameters. The images were processed using the Nanoscope Analysis v. 1.50 (Bruker Co.) software.

### Estimation of cell volume

Cell volume was estimated by optical microscopy and analysis of images collected every fifth cell budding during the routine procedure of determining the reproductive potential. The images were captured with the Nikon Eclipse E200 microscope equipped with the Olympus DP26 digital camera. Cell diameter (d) was measured using the Olympus cellSens Standard software in various planes for each cell and the mean value was used for calculations. Assuming that each cell has a regular shape similar to the sphere, the cell volume (V) was calculated as V = 4/3π (d/2)^3^.

### Phenotypic analysis—a spot test for sensitivity to Congo red, Calcafluor White, MMS and sodium chloride

Yeast cultures were grown to exponential phase (OD_600nm_ between 0.8 and 1) and serially diluted to different cellular concentrations as indicated. Five microliters of each cell suspension was spotted onto agar plates containing various concentrations of Congo red (Sigma-Aldrich), Calcafluor White (Sigma-Aldrich), methyl methanesulfonate (Sigma-Aldrich) and sodium chloride (Sigma-Aldrich). Growth was registered 48 h after incubation at 30 °C. All phenotypes described in this work were confirmed by multiple tests.

### Statistical analysis

The results represent the mean ± SD values for all cells tested in two independent experiments (80 cells). The differences between the mutant strain compared to the wild-type strain were estimated using a one-way ANOVA and Dunnett’s post hoc test. The values were considered significant if *p* < 0.01. Statistical analysis was performed using the Statistica 10 software.

## Results

### The budding lifespan of the cell wall mutants

**Fig. 1:**
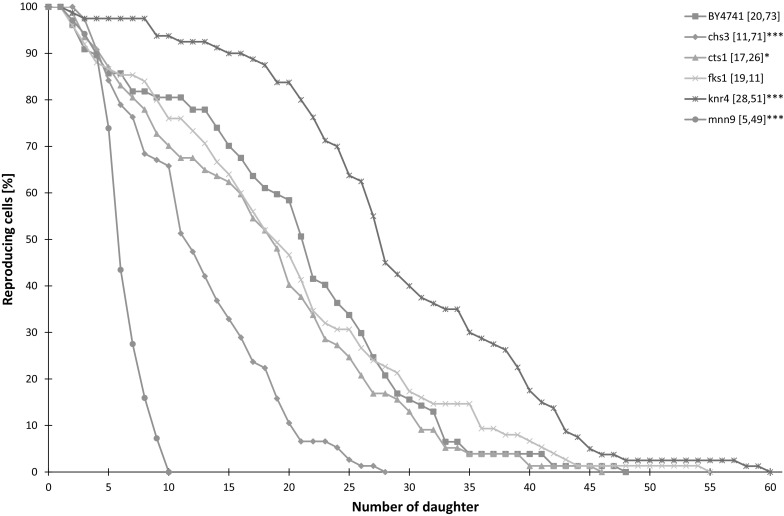
Comparison of the reproductive potential of the haploid wild type yeast strain BY4741 and isogenic mutant strains *chs3Δ*, *cts1Δ*, *fks1Δ*, *knr4Δ*, *mnn9Δ.* Mean values (for total 80 cells from two independent experiments) of the reproductive potential are shown in parentheses

To obtain insight into the role of cell wall in aging of the *S. cerevisiae* yeast, we examined strains that were impaired in the process of cell wall synthesis. Initially, we analysed the budding lifespan measured as the number of daughter cells produced by the mother cell. As seen in Fig. 1, disturbances in the regulation of cell wall synthesis had a significant impact on the budding lifespan. Cell wall mutations considerably altered cell lifespan at the exponential growth phase, significantly increasing (*knr4Δ*) or decreasing (*cts1Δ*, *chs3Δ* and *mnn9Δ*) the lifespan compared to the BY4741 strain (Fig. 1). Only the impairment in 1,3-β-glucan synthase activity (*fks1Δ*) did not affect the budding lifespan. The most dramatic reduction of the trait (almost 4 times compared with BY4741) was observed for the *mnn9Δ* mutant defective in protein mannosylation. Moderate impact of the cell wall mutant on the budding lifespan was observed for *knr4Δ*, *chs3Δ* and *cts1Δ* strains. Interestingly, more than half the population of *chs3Δ* exploded during a routine procedure determining the reproductive potential (data not shown). Thus, knock-out of the nonessential function of cell wall-related genes had an effect on the budding lifespan (Fig. 1).

### Changes in the cell volume during budding lifespan

**Fig. 2:**
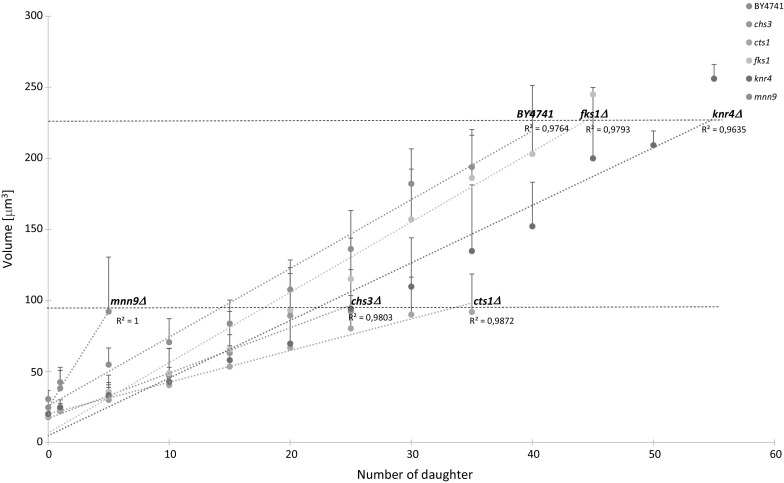
Dependence of cell volume on the number of daughters accomplished by mother yeast cells. The results represent values for all cells tested in two independent experiments (80 cells). The bars indicate SD

Next, we tested changes in cell volume during subsequent cycles of the mutant and wild-type strains. In view of the hotly debated influence of cell volume on the budding lifespan, we obtained data which may throw new light on the hypertrophy hypothesis. Our data show that the basic assumptions of the hypothesis may not be obvious. As seen in Fig. 2, the *fks1Δ* and *knr4Δ* mutants achieved a similar maximum volume to that of the wild-type strain when they finished budding. However, in the case of *chs3Δ*, *cts1Δ* and *mnn9Δ*, the maximum volume was about 50% lower compared to the wild-type strain. Consequently, for this group of strains, a higher cell volume was associated with a relatively higher budding lifespan.

### Estimation of the cell wall thickness determined by TEM

**Fig. 3:**
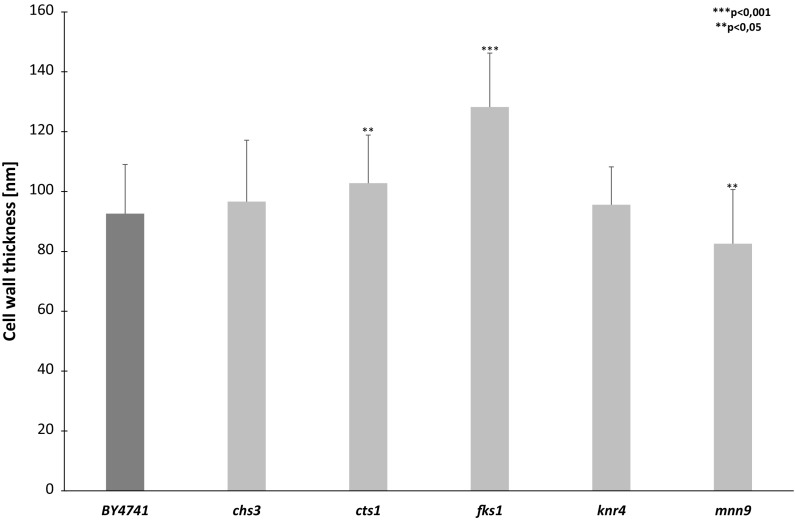
Comparison of cell wall thickness between BY4741 (wild-type) and cell wall mutants (*chs3Δ*, *cts1Δ*, *fks1Δ*, *knr4Δ* and *mnn9Δ*) measured in the images from the transmission electron microscopy using the EM Menu4 software. The means were obtained for 15 cells. The bars indicate SD. Statistical significances were assessed using ANOVA and the Dunnett’s post hoc test (**p < 0.05, ***p < 0.001)

Attempting to explain these changes in terms of cell wall properties, we analysed several morphological parameters of the wall, including thickness, roughness, and Young’s modulus as well as cell volume. Cell wall thickness determined on the basis of the electron microscope measurements did not vary in *mnn9Δ*, *knr4Δ* and *chs3Δ* but increased in the *cts1Δ* and *fks1Δ* strains compared to wild-type cells (Fig. 3). However, the lifespan parameter did not correlate with cell wall thickness. The *mnn9Δ* mutant displaying a dramatically reduced lifespan showed similar wall thickness as wild-type, while the highest increase in the cell wall thickness was observed in the *fks1Δ* mutant in which the lifespan was not affected.

### Cell wall surface characterisation

**Fig. 4:**
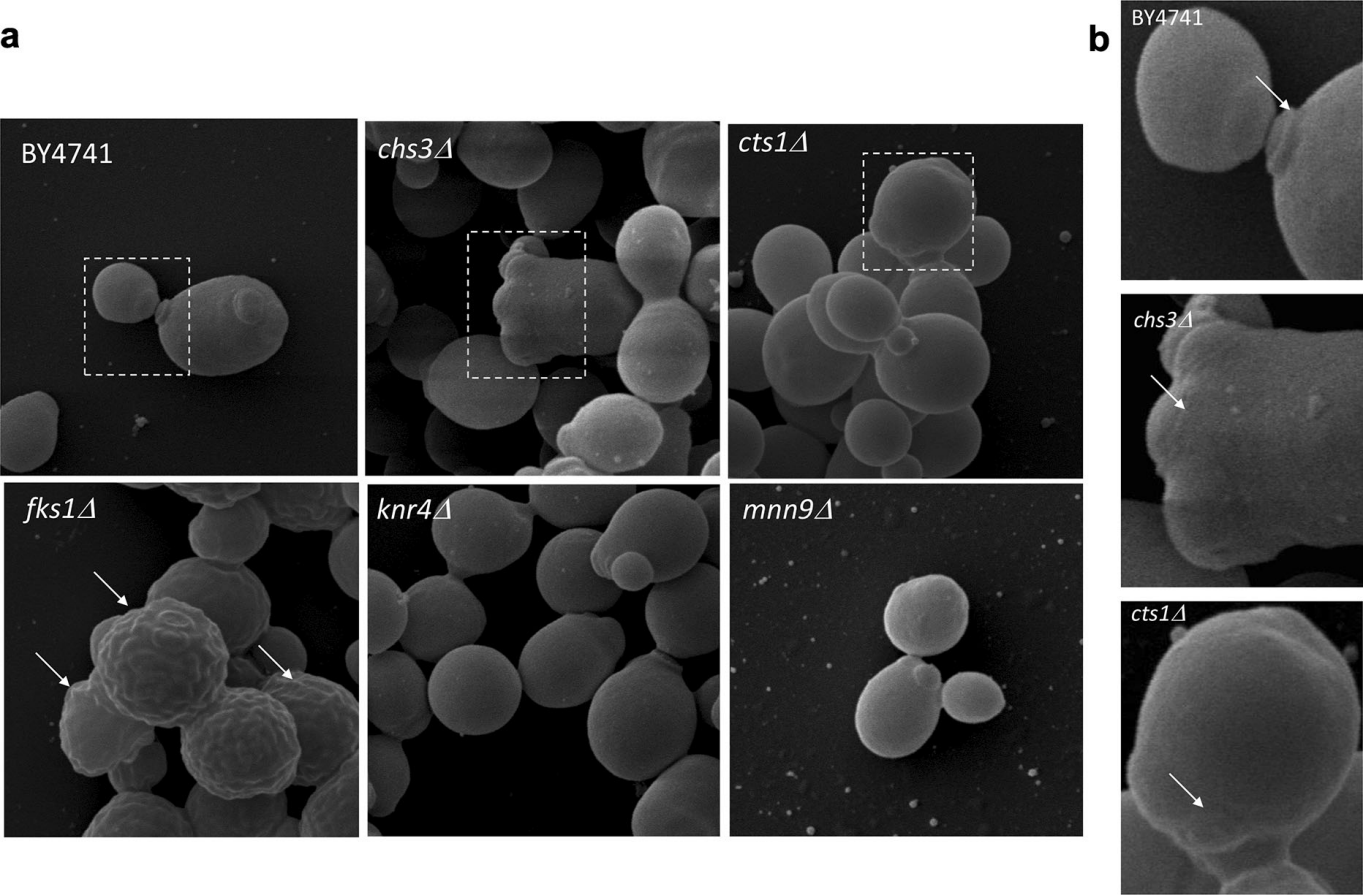
Scanning electron microscopy images of *Saccharomyces cerevisiae* cells for the BY4741 wild-type strain and cell wall mutants: *chs3Δ*, *cts1Δ*, *fks1Δ*, *knr4Δ*, and *mnn9Δ*. The arrows in **a** indicate wrinkles on the cell wall surface observed in *fks1Δ*. The arrows in **b** indicate changes in bud scars structure occurring in the *chs3Δ* and *cts1Δ* mutants compared to wild-type

**Fig. 5:**
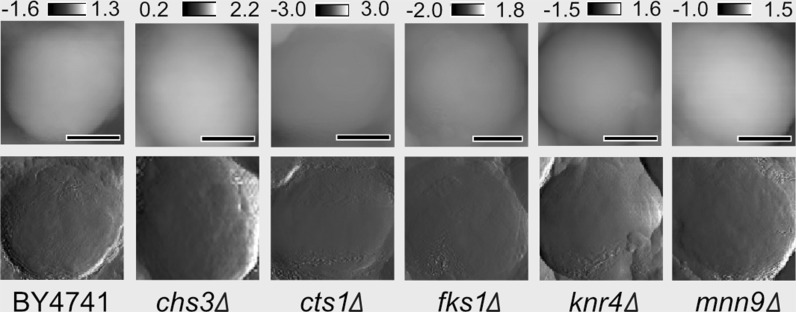
Representative AFM images of *Saccharomyces cerevisiae* cell surface for the wild type strain BY4741 and five cell wall mutants: *chs3Δ*, *cts1Δ*, *fks1Δ*, *knr4Δ* and *mnn9Δ*. The upper array of photos was collected in the Height mode. The height scale above the photos is given in μm. The corresponding lower array of images was obtained in the Peak Force Error mode. The scale bar = 1 μm

For inspection of possible deformations in the cell wall surface, e.g. collapse, protrusions or breakage, we used scanning electron microscopy (SEM) and atomic force microscopy (AFM) as two complementary tools. SEM micrographs of wild type cells and cell wall mutants are given in Fig. 4. Cells of the mutants differed to some extent in shape, volume and bud scar morphology compared to the wild-type strain. The most striking differences in the wall topography were observed for *fks1Δ* cells, which showed some protrusions in the form of wrinkles. We also noticed that the *chs3Δ* and *cts1Δ* mutants had abnormal bud scarring (Fig. 4b). The AFM imaging did not show clear modifications of cell wall topography in the examined mutants at the exponential phase of growth (Fig. 5). Representative images collected in the Height mode are given in the upper row, while corresponding images obtained in the Peak Force mode are provided beneath. The latter mode is particularly sensitive to homogeneities in the wall material, especially those that are related to the mechanical surface properties. Interestingly, the presence of bulges and protuberances on the surface that could be seen in SEM micrographs for some *fks1Δ* cells was not confirmed in the AFM imaging in either the Height or the Peak Force modes. This may suggest possibility of their incidental occurrence or formation resulting of specific sample preparation.

Further, we analysed a possible relationship between elastic properties of the cell wall and the lifespan parameter. Young’s modulus (E) of cell wall obtained through nanoindentation was taken from the literature (Dague et al. 2010). This parameter was generally lower in the examined mutants compared to the BY4741 strain, with the exception of the *mnn9Δ* strain. Therefore, the most dramatic reduction of the reproductive potential observed for the *mnn9Δ* cells was not reflected in the changes of the modulus. Moreover, reduction in E reported for the *knr4Δ* and *fks1Δ* strains corresponded both to the increased and non-altered lifespans, respectively. Cell wall roughness reported in literature (Dague et al. 2010) was generally much higher in the mutants compared to BY4741, with the exception of *fks1Δ*. The *fks1Δ* mutant showed a reproductive potential similar to that of the wild-type strain, while the *mnn9Δ* mutant, characterised by the highest increase in roughness, displayed the lowest reproductive potential among the strains. In turn, increase in roughness reported for the *chs3Δ* and *knr4Δ* strains corresponded to the reduced and extended reproductive potentials, respectively. Taken altogether, these data indicate lack of clear association between the reproductive potential and roughness and elastic properties of the wall surface.

### Sensitivity to agents disturbing the wall biosynthesis

**Fig. 6:**
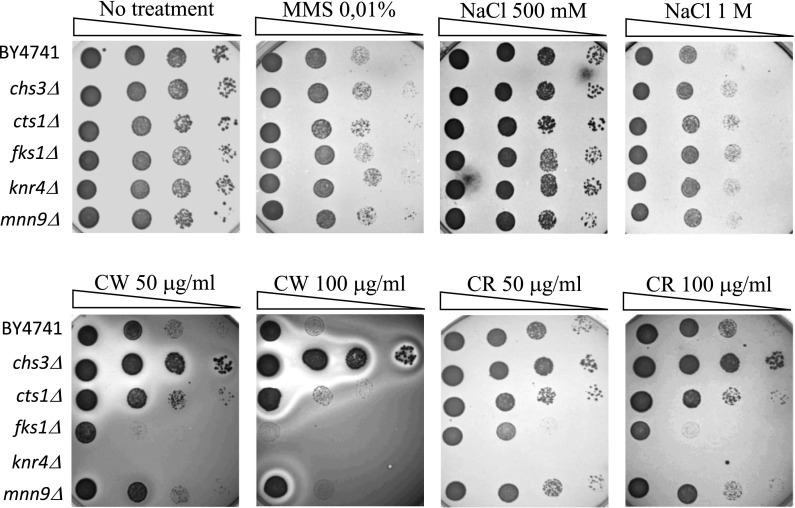
Sensitivity of the BY4741 strain and cell wall mutants with disrupted *CHS3*, *CTS1*, *FKS1*, *KNR4*, *MNN9* genes to Calcofluor white (CW), Congo red (CR), Methyl methanesulfonate (MMS) and sodium chloride (NaCl). Yeast strains were grown in YPD medium (28 °C), spotted onto YPD plates containing the indicated amounts of CW, CR, MMS, NaCl and incubated at 28 °C. Growth on YPD agar plates was treated as a control. Representative results from two independent experiments are shown

Finally, to examine whether the mutants truly had defects in cell wall biosynthesis, we conducted spotted tests on media containing Calcofluor white (CW) or Congo red (CR). Both are known to disturb cell wall biosynthesis in normal strains. The *fks1Δ* and *knr4Δ* strains were hypersensitive to CW and CR, and the *fks1Δ* strain appeared to be slightly less sensitive than *knr4Δ*. Additionally, the *chs3Δ* mutant was hyper-resistant to both CW and CR. Moreover, the impairment of the analysed cell wall-related genes did not affect the growth of the cells under methylation agent (0.01% Methyl methanesulfonate) and osmotic stress (0.5 M and 1 M NaCl) (Fig. 6).

## Discussion

Involvement of the *S. cerevisiae* yeast cell wall in cell growth and reproduction, e.g. formation of septum and mother–daughter cells separation, is quite well understood (reviewed by Cabib et al. 2001; Roncero and Sanchez 2010) However, the relationship between composition, molecular organisation and physicochemical properties of the wall and cell longevity has not been studied extensively so far. To address that problem, we have examined the effect of several wall mutations, including *chs3Δ*, *cts1Δ*, *fks1Δ*, *knr4Δ* and *mnn9Δ*, on the budding lifespan of yeast cells. Both the deposition of primary and secondary septa as well as disassembly of the structures engages the machinery of cell wall synthesis and decomposition, which is typically employed into formation and remodelling of the lateral cell wall.

In earlier studies, which attempted to link aging to septation, the focus was given to bud scar characteristics. Bud scars provide a means of determining the reproductive age of cells (Barton 1950; Egilmez et al. 1990; Sinclair et al. 1997). Mortimer and Johnston (1959) suggest that accumulation of chitin (in bud scars) may limit budding. Later data disproved that theory and demonstrated that scarring is a result rather than cause of replicative aging (Egilmez and Jazwinski 1989). More recent data suggest that chitin scar breaks during aging, which may result from the bud scarring’s reduced capacity of stretching despite chitin network elasticity (Powell et al. 2003).

Interestingly enough, our study showed that strains defective in genes involved in wall synthesis or remodelling may either increase or decrease the budding lifespan parameter to various degrees. Exceptionally, glucan synthesis impairment (*fks1Δ*) had no effect on lifespan. The Fks1 protein is a catalytic subunit of the β-1,3-d-glucan synthase involved in polymerisation of the main structural polysaccharide, which may represent up to 80% of the dried wall weight, and takes part in wall synthesis, maintenance and remodelling (Utsugi et al. 2002). *S. cerevisiae* has three glucan synthase genes but Fks1p functions as the major β-1,3-glucan synthase during vegetative growth in yeast (Klis et al. 2006). It is, however, one of functionally alternative subunits of the β-1,3-d-glucan synthase complex. The other protein, Gsc2/Fks2, may repossess the main function in polymerisation of the glucan depending on the environmental conditions (Klis et al. 2002). Fks1p is directly involved in the budding process, being responsible for deposition of β-1,3-d-glucans during formation of the secondary septa. Although the mutation did not alter the lifespan and the final cell volume, the wall formed by the mother cells was thicker relative to the wild-type strain according to our TEM measurements, and of reduced stiffness and increased roughness at the stationary stage as reported by Dague et al. (2010).

The majority of the examined mutants, including *cts1Δ*, *chs3Δ*, and *mnn9Δ*, decreased the budding lifespan. Moderate reduction in the lifespan was observed for *chs3Δ* and *cts1Δ* mutants, which were defective in chitin biosynthesis and chitinase activity, respectively. Chitin is a compound that represents only 1–2% of the dry weight of wall in normal growth but may show considerably enhanced levels in response to the altered environment or altered wall properties due to genetic disturbances (Carotti et al. 2002; Klis et al. 2006; Popolo et al. 1997). Despite low abundance of chitin, the interlinks established between glucans and also proteins provide the wall with appropriate strength and structural integrity (Klis et al. 2006; Orlean 2012). The CHS3 comprises a transmembrane catalytic unit responsible for a great majority of chitin synthesis during normal growth and is involved in the chitin stress response (Bulik et al. 2003). It also takes part in the deposition of the chitin-containing ring at the base of the emerging bud (Shaw et al. 1991). Moreover, Chs3p along with Fks1p is involved in the deposition of secondary septa on both daughter and mother sides (Ortiz and Novick 2006). Although Chs3p is vital for septation, absence of the catalysing unit may be compensated by other chitin synthetases and the *chs3Δ* mutant produces a modified three-layered septum (Shaw et al. 1991). The chitin synthetase 3 impairment was related to the reduced volume of the final cell without changes in wall thickness. The AFM measurements reported by Dague et al. (2010) showed an increase in the wall surface roughness and a reduction in Young’s modulus determined by indentation with a nano probe (Dague et al. 2010).

The chitin accumulation through the lifespan mostly as the ring structure at the budding neck was proposed as a possible constraint of longevity in yeast due to decrease in the available wall space for subsequent cell divisions (Mortimer and Johnston 1959), however, this suggestion has not been supported with experimental studies (Powell et al. 2003). The number of chitin bud scars indeed increases in the course of subsequent cell cycles (Egilmez et al. 1990) and is therefore considered a biomarker for replicative cell age (Powell et al. 2003). The scar tissue is flexible and may undergo some stretching as a result of cell expansion (Powell et al. 2003). However, this does not result in local discontinuity since the inelastic chitin ring undergoes breakage that is symmetrically distributed (Powell et al. 2003). The local damage is also prevented by tight founding of the chitin with β-1,3-glucan in the cell wall (Arroyo and Arroyo 2013).

The *cts1Δ* mutant’s budding lifespan was much lower compared to *chs3Δ* and both strains were characterised by reduction in the final cell volume and some insignificant changes in wall thickness. Cts1 chitinase is one of essential enzymes, apart from a number of glucanases/glucanosyl transferases, needed for septa material degradation to enable mother and daughter cell separation. Since the septa is digested exclusively from the daughter’s side, the mother cell remains after the cells dissociation with a remnant bud scar. This process of septa destruction is highly controlled to maintain integrity of the separated daughter cell, and therefore deletion of *CTS1* may considerably affect the process of proliferation. Both chitin synthetase (*chs3Δ*) and chitinase (*cts1Δ*) mutants showed changes in the birth scar morphology as revealed in the SEM micrographs. These observations support earlier reports indicating their specific activity in the region of septum formation and mother–daughter cell separation.

The literature data (Dague et al. 2010) indicate a considerable decrease in wall stiffness and increase in wall surface roughness both at the exponential and stationary stages.

Cts1p plays a particularly important role in the digestion/degradation of the primary septum that is required for separation of mother and daughter cells. The *CTS1* gene deletion may substantially affect this process. Although both enzymes are essential for formation of the septa, where Chs3 contributes mainly to structural reinforcing, some compensatory mechanisms may be activated if chitin is not produced at this location (Schmidt 2004). However, it provides a rather minor direct contribution to the overall chitin synthesis and chitin content level in normal conditions. It is mostly implicated in the formation of the secondary septum specifically required for mother–daughter cell separation during the budding process. This interaction seems to explain the increase in wall surface roughness and reduction in Young’s modulus determined by indentation with the atomic force microscopy probe (Dague et al. 2010). We could see that the mutations affecting the mother–daughter cell separation reduced the mother cell’s longevity. Both mutants showed changes in the birth scar morphology as revealed in this study by SEM micrographs. In addition, these mutations reduced the final volume of mother cells but did not alter the cell wall thickness markedly.

The most dramatic reduction in the budding lifespan was observed for the *mnn9Δ* mutant defective in the synthesis of the α-1,6-mannan. The Mnn9 protein is a subunit of the mannosyltransferase complex located in Golgi membranes which takes part in the synthesis of the mannan backbone *N*-linked to the cell wall proteins (Yip et al. 1994). Glycosylated proteins are the main compound of the outer wall layer in fungi and the extent and pattern of protein glycosylation is essential for such cell functions as morphogenesis, wall adhesive properties and wall permeability (Orlean 2012). Due to a relatively high content of the compound in cell wall any disturbance in its biosynthesis must affect the wall’s integrity. Additionally, this mutation may affect directly the budding longevity through generation of abnormalities in the septum, which in the case of *mnn9Δ* is much thinner or shows signs of repair or remediation (Schmidt et al. 2005). The cell wall thickness and the rigidity of the mutant were unchanged, probably because the defect in the mannosylation was compensated by a many-fold increase in the chitin content and a slight increase in the amount of glucans (Dague et al. 2010). The mutation inhibited cell volumetric growth at budding; however, it dramatically increased wall permeability and wall surface roughness.

Extended cell longevity was observed exclusively for the *knr4Δ* mutant. The Knr4 protein is a transcriptional regulator of cell wall chitin and glucan synthesis (Hong et al. 1994). In this way Knr4p is involved in coordinating cell cycle advancement with cell wall integrity. The mutant showed a slight increase in cell wall rigidity as revealed by AFM probe indentation and a marked increase in wall surface roughness in the exponential growth stage. The latter could be the result of an increase in the mannan content (Dague et al. 2010). The improvement in the mechanical properties, despite decrease in the β-glucan content, might be explained by the marked increase in chitin content (Hong et al. 1994) that could contribute to establishing more abundant wall polymer interlinking.

As asexual reproduction, budding is unavoidably associated with a continuous growth of the mother cell’s volume (Molon and Zadrag-Tecza 2016a; Zadrag-Tecza et al. 2009). Therefore, we subsequently attempted to address the changes in budding lifespan referring to the volume which the cell may achieve in the case of a given wall mutation, and thus verify the hypertrophy hypothesis that has been hotly debated recently (Bilinski and Bartosz 2006; Ganley et al. 2012; Kaeberlein 2012; Wright et al. 2013; Yang et al. 2011). This hypothesis assumes that cell volume is a major factor determining finished budding (Bilinski and Bartosz 2006; Bilinski et al. 2012). Further papers suggest that the rate of volume growth is a factor regulating the parameter (Yang et al. 2011). In support of the hypertrophy hypothesis, studies on various mutations (Yang et al. 2011) and nutrition conditions (Turner et al. 2012) were reported. There is also some evidence against the argument. Moretto et al. (2013) showed that the knock-out *FOB1* gene increases budding lifespan without altering cell volume (Moretto et al. 2013). Additionally, diploid yeast has larger cells than haploid, and shows an increased lifespan compared to the haploid cells (Kaeberlein et al. 2005). Latest data suggest that yeast cells can achieve a significantly higher volume than the wild-type strain (Molon and Zadrag-Tecza 2016a). In turn, while exploring the cell wall mutants, we show here that yeast cells gain a 50% smaller maximum volume than the wild-type strain. What is interesting, in the case of the *cts1Δ*, *chs3Δ* and *mnn9Δ* mutants the cells achieved various budding lifespans for the same cell volumes. In our other studies (Molon and Zadrag-Tecza 2016a), cells of some mutants (*sfp1Δ*) showing decreased budding lifespan reached dramatically enhanced volumes compared to the wild-type strain. Therefore, cells may display a wide range of volume in the same genetic backgrounds and under the same environmental conditions when they stop budding. To sum up, our data and all of the aforementioned reports support the conviction that achieving hypertrophy cannot be the only factor that determines the budding lifespan of the cell. Impact of hypertrophy on aging requires therefore further studies and clarification.

Using multiple imaging techniques, e.g. SEM, TEM and AFM, and the spoil test, as well as taking into consideration indentation measurements of wall stiffness reported in literature (Dague et al. 2010), we attempted to establish a putative association between the phenotypic modifications in the cell wall and the changes in the budding lifespan. However, we did not find any clear association between cell wall characteristics or cell morphological traits and the budding lifespan, even though the disturbances in wall biosynthesis had a pronounced effect on both the lifespan and morphology of cells. This may be a consequence of extended molecular and biochemical adaptive responses of cells to the point mutations in cell wall biosynthesis. The response could be driven by sensing of the wall mechanical status and activation of the cell wall integrity (CWI) pathway (Levin 2011), which affects multiple cellular processes. It may, among other things, induce a complex compensatory alteration of cell wall composition, e.g. hyperaccumulation of chitin (Popolo et al. 1997; Ram et al. 1998), which may have impact on the septation process and the mother–daughter cell separation. In addition, more recent studies have indicated that the mitogen-activated protein kinase (MAPK)-mediated signalling pathway activated by cell wall stressors affects the cell cycle progression (Carbo and Perez-Martin 2010) which may influence the budding lifespan.

The above may suggest, therefore, that establishing a cross talk between the CWI pathway and the signalling networks controlling the aging process might provide a better understanding of the complex mechanism of the budding lifespan.
